# Biomechanics of aortic valve annuloplasty: Same goal, different techniques

**DOI:** 10.1016/j.xjtc.2024.03.004

**Published:** 2024-03-14

**Authors:** Evaldas Girdauskas, Theresa Holst, Sina Stock, Thomas Kröncke, Maria von Stumm, Josua A. Decker

**Affiliations:** a Department of Cardiothoracic Surgery, University Hospital Augsburg, Augsburg, Germany; b Department of Diagnostic and Interventional Radiology, University Hospital Augsburg, Augsburg, Germany; c Department of Congenital and Pediatric Cardiac Surgery, German Heart Center Munich, Technische Universität München, Munich, Germany; d Division of Congenital and Pediatric Heart Surgery, University Hospital Munich, Ludwids Maximilians University, Munich, Germany

**Figure:**
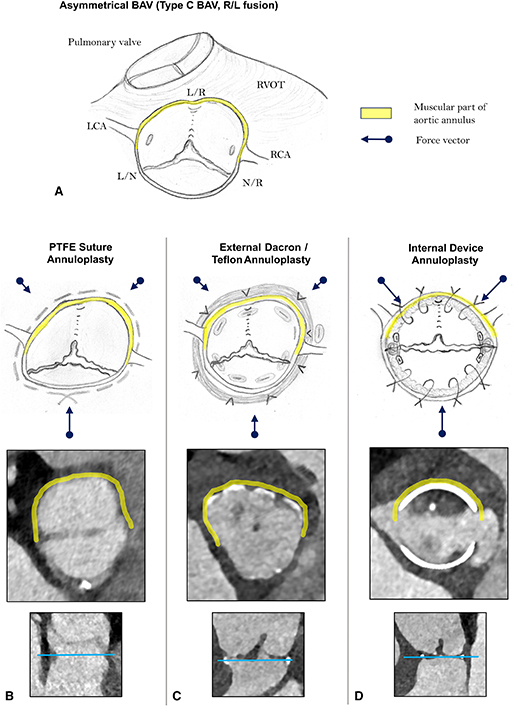
Biomechanical effects of 3 different aortic valve annuloplasty approaches.

**Central Message**

Different annuloplasty techniques in bicuspid aortic valve repair exert specific biomechanical forces on aortic annulus components and thus have different influences on geometric annulus remodeling.

Aortic valve (AV) repair has become an established technique in the nonelderly adults presenting with aortic regurgitation, as demonstrated by very satisfactory periprocedural and 1-year cardiac event-free survival in the multicenter GARY registry (The German Aortic Valve Registry).^1^ Furthermore, AV repair was associated with significantly better 1-year survival and 1-year cardiac event-free survival compared with surgical aortic valve replacement in propensity score-weighted analysis of patients with aortic regurgitation.^2^ Therefore, a broader adoption of AV repair techniques, in particular in young patients with a congenital bicuspid AV (BAV) disease, seems highly warranted.

Annuloplasty is a crucial component of BAV repair with significant implications on the durability of the repair.^3^ Various annuloplasty techniques have been proposed over recent decades; all of them strive for the same goal of aortic annulus remodeling and stabilization. However, biomechanical principles and dynamic effects of annuloplasty techniques on the aortic annulus have been address only in a rudimentary manner. Quite a number of monocentric studies comparing the outcomes of different AV annuloplasty concepts have been published^4^^,^^5^; however, no multicenter and prospective comparative randomized trial on this topic is available. Understanding the biomechanical implications of any specific annuloplasty method is crucial to identifying technical shortcomings and limitations.

From a pathophysiological point of view, active remodeling of the rigid muscular aortic valve annulus^E1^ and the restoration of a symmetric postrepair BAV configuration^E2^ are the key components of a durable BAV annuloplasty. In Figure 1, we highlight the biomechanical aspects of 3 established BAV annuloplasty approaches. Specific focus was on the effects of different annuloplasty techniques on the geometric shape of the annulus in relation to the symmetry of the commissural orientation.

**Figure 1:**
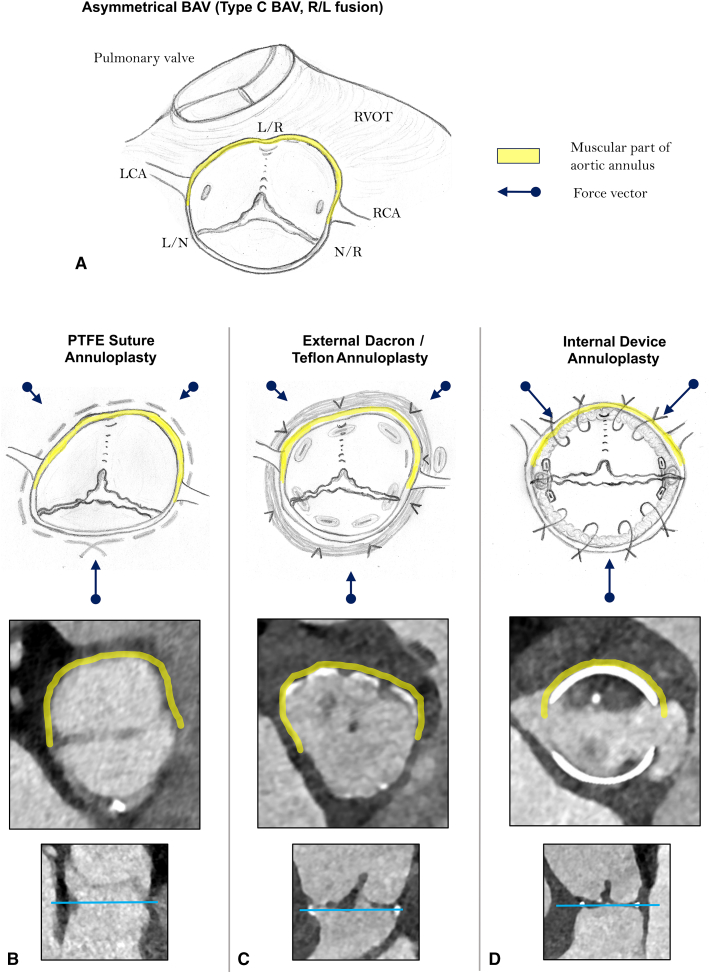
Biomechanical effects of 3 different aortic valve annuloplasty approaches. A, Asymmetrical bicuspid aortic valve (type C, right-left fusion). B, Polytetrafluoroethylene suture annuloplasty. C, External Dacron/Teflon annuloplasty. D, Internal device annuloplasty.

As demonstrated in Figure 1, *A*, the aortic annulus consists of 2 fundamentally different anatomic components: a flexible and unsupported fibrous aortic annulus, which extends from the right noncoronary commissure to the left fibrous trigone; and a rigid and firmly embedded muscular aortic annulus that is largely covered by the right ventricular outflow tract. There are some supportive data on the different biomechanical features of the muscular versus fibrous AV annulus. Our preliminary segmental AV annulus analysis by means of regional longitudinal strain (RLS) revealed significantly decreased RLS in the muscular part of the AV annulus in patients with aortic regurgitation versus healthy controls, whereas RLS values were comparable in the fibrous component of the AV annulus.^E1^ Furthermore, Benhassen and colleagues^E3^ used sonomicrometry crystals for the evaluation of segmental AV annulus dynamics during the cardiac cycle, and convincingly showed significant differences in the segmental annulus deformation between the right coronary versus the noncoronary sinus.

Based on our previous clinical observations^E4^ and some supportive data from the literature,^E1^ we argue that both aortic annular components behave differently in our attempts to reduce and remodel the annulus diameter. The muscular aortic annulus, in particular in the area between the left and right coronary commissure and the midpart of the right coronary sinus, is deeply anchored in the interventricular muscular septum and, therefore, is much less amenable to geometric reshaping maneuvers. In line with this statement, an experimental study by Benhassen and colleagues^E1^ revealed significant differences in the AV annulus dynamics after external Dacron prosthesis annuloplasty versus polytetrafluoroethylene (PTFE) suture annuloplasty.^E1^

The majority of BAVs are congenitally asymmetric; that is, type B or C BAV morphotype (previously type I Sievers)^E5^ and present in the form of right and left coronary cusp fusion (Figure 1, *A*). In such cases, the proportion of the muscular aortic annulus outweighs substantially the fibrous annular component, and therefore, extensive geometric reshaping of the muscular aortic annulus is needed to obtain a symmetric postrepair BAV configuration.

Taking these considerations into account, an aortic annulus reduction with a circular suture annuloplasty (Figure 1, *B*) acts predominantly on the area of the lowest tissue resistance; that is, in the part of the unsupported fibrous aortic annulus component. The main force vectors are directed toward the muscular interventricular septum; that is, the PTFE suture pulls the fibrous component of the aortic annulus toward the muscular septum. The reshaping of the muscular aortic annulus is incomplete and the asymmetrical BAV configuration persists after the suture annuloplasty resulting in markedly restricted fused cusp mobility and increased transvalvular gradients (see Table 1). Previous data indicate a significant amount of recurrent aortic regurgitation and number of redo surgery cases after PTFE suture annuloplasty.^E4^

**Table 1:** Influence of the annuloplasty technique on functional result after repair of severely asymmetric bicuspid aortic valve (type C)

Annuloplasty	PTFE suture	External prosthesis	Internal ring
Commissural orientation after repair	Very asymmetric (type C)	Asymmetric (type B)	Symmetric (type A)
Systolic opening of the fused cusp	Restricted	Restricted	Normal
Systolic transvalvular gradients	Increased (dpmean > 15 mm Hg)	Increased (dpmean 10-15 mm Hg)	Normal (dpmean <10 mm Hg)

*dpmean*, Mean transvalvular pressure gradient.

External prosthesis annuloplasty (ie, Dacron graft or Teflon strip) aims to circularly reduce aortic annulus diameter and entails several fixation points in the fibrous and muscular annular components (Figure 1, *C*). The force vectors act more homogeneously on the aortic annulus, compared with the suture annuloplasty. However, active geometric reshaping of the muscular annulus is also limited by the heterogeneous aortic annular tissue characteristics, in particular in the midpart of the right coronary sinus. In other words, annular reduction occurs predominantly in the regions of lower annular tissue resistance (ie, fibrous component). Furthermore, the asymmetrical annular shape in type C BAV is not sufficiently corrected by external prosthesis annuloplasty, resulting in persisting asymmetry after AV repair (Figure 1, *C*). As a consequence, the mobility of the fused cusp is frequently limited and transvalvular gradients increased after external prosthesis annuloplasty (see Table 1).

Internal ring annuloplasty with orientation of commissural posts at 180° (eg, HAART 200 device, CorCym) (Figure 1, *D*) enables selective reshaping of both annular components by forcing them into a strictly symmetric configuration. An active adjustment of both annular components to the internal device shape occurs, causing an extensive reduction of the muscular annular portion. The intraoperative sizing for internal ring annuloplasty using HAART 200 device is based on the geometric orientation and size of the nonfused cusp. Specific ball sizing is used for the measurement of the nonfused cusp to assess the commissural orientation and, in particular, surface area of the nonfused cusp. This sizing maneuver generally provides the values of 23 or 25 mm; all remaining numbers are unusual. The fused cusp and the muscular component of the AV annulus, respectively, are actively adjusted to the size of the nonfused (ie, fibrous part of the annulus) during the internal ring implantation, resulting in a symmetric geometric shape of repaired BAV. In other words, the size of the nonfused cusp (ie, length of the fibrous AV annulus) defines the postrepair length of the muscular annulus, which is required to obtain a completely symmetrical BAV configuration. Consequently, a completely symmetrical postrepair BAV shape is restored, allowing for better fused cusp mobility and lower transvalvular gradients (see Table 1).^E2^

Considering the biomechanical differences, we advocate prospective comparative outcome studies among different annuloplasty approaches.

## Conflict of Interest Statement

The authors reported no conflicts of interest.

The *Journal* policy requires editors and reviewers to disclose conflicts of interest and to decline handling manuscripts for which they may have a conflict of interest. The editors and reviewers of this article have no conflicts of interest.
